# Developing the marine medicine syllabus for medical sciences students: a multiphase design study

**DOI:** 10.1186/s12909-023-04461-4

**Published:** 2023-06-23

**Authors:** Farshad Heydari, Mohammad Nobakht, Amir Vahedian-Azimi, Seyed Shahram Mirzamani, Seyed Tayeb Moradian, Abbas Ebadi, Morteza Kameli Morandini

**Affiliations:** 1 Marine Medicine Research Center, Baqiyatallah University of Medical Sciences, Tehran, Iran; 2 Trauma Research Centre, Nursing Faculty, Baqiyatallah University of Medical Sciences, Tehran, Iran; 3 Strategic Navy Force of the Army of Islamic Republic of Iran, Faculty of Convergent Science and Technology, Science and Research Branch, Islamic Azad University, Tehran, Iran; 4 Atherosclerosis Research Center and Faculty of Nursing, Baqiyatallah University of Medical Sciences, Tehran, Iran; 5 Behavioral Sciences Research Center, LifeStyle Institute, Baqiyatallah University of Medical Sciences, Tehran, Iran; 6 Nursing Faculty, Baqiyatallah University of Medical Sciences, Tehran, Iran

**Keywords:** Marine, Maritime, Medicine, Medical, Education, Syllabus

## Abstract

**Background:**

Marine medicine is one of the medical fields that deals with the health and safety of people related to the sea but the marine medicine syllabus for education to the students is not specified yet. The present study aimed to develop the marine medicine syllabus to medical sciences students education.

**Methods:**

This study was conducted in three phases. First, a literature review was conducted to find the concepts and topics related to marine medicine. Second, a content analysis research method was conducted. Data collection was done first by using semi-structured interviews with the 12 experts in marine medicine. Sampling was purposeful and continued until data saturation was reached. The information obtained from the interviews was analyzed by conventional content analysis with Geranheim's method. The found topics in the literature review and content analysis of interviews were combined and formed the initial draft of the marine medicine syllabus, which was validated with the Delphi method in the third phase. The Delphi was conducted in two rounds and the panel consisted of 18 experts in the field of marine medicine. After the completion of each round, the items that had less than 80% consensus among the participants were removed and the remaining topics after round two formed the final syllabus of the marine medicine.

**Results:**

The findings showed that the marine medicine syllabus should include an overview of marine medicine, health at sea, common physical diseases and injuries at sea, subsurface medicine and hyperbaric, safety action in marine incidents, medical care at sea, psychology at sea and medical examinations of people working at sea main topics and their sub topics.

**Conclusions:**

Marine medicine is an extent and specialized medical field which has been neglected and it is necessary to teach this lesson to medical sciences students with the syllabus found in the present study.

## Background

The sea is the cradle of life and life is born from seas and oceans [1]. Oceans are one of the most important and largest environments on our planet, which provide almost half of the oxygen needed for life [2]. However, when considering occupational health and safety issues and the availability of medical care, the oceans are the most dangerous workplaces on the planet [3]. Medical care for sick and injured people at sea is challenging because its scope is wide and the problems faced at sea are specific and it creates problems in assessment, treatment and providing care [4]. Maritime medicine refers to any medical activity related to employment, working conditions, living conditions, health and safety of employees at sea. These employees can be employees of the merchant fleet, navy, fishing vessels, marine pilots, shore installations, students of maritime schools and yachts [5].

Today, there is an international postgraduate course in maritime medicine, but the requirements of this course differ from country to country. These courses are usually organized locally, and different courses are without specific standards and have different content. Progress has been made in this field in recent years, and the Master's level of this course has been made available at the University of Cadiz in England and Spain, as well as at the Diploma level at the University of Brest in France. In Poland, marine and tropical medicine is taught at the postgraduate level [6]. In Denmark, marine doctors are also general practitioners who have received special training in marine medicine. This special education is also seen in Denmark's neighboring countries. These doctors perform mandatory medical examinations for seafarers, fishermen and coastal workers [7]. However, there has been a long-standing disagreement and debate regarding the educational content provided to marine doctors [5].

The educational content that should be taught in the curriculum is called the syllabus. Syllabus is a contract between the instructor and the learner, a tool for expressing responsibility and commitment, a guide for the instructor, and they ensure mutual and correct understanding between the instructor and the learner and minimize ambiguity in the course. Selecting and arranging the appropriate educational content is necessary to achieve a successful education [8]. Advances in medical knowledge and changes in the skills and attitudes that learners will need to perform their careers result in the major changes in medical curriculum and syllabus worldwide, and there is still a need for redesigning and reforming medical education [9].

Therefore, considering Iran as a marine country that is bounded from the north by the world's largest lake and from the south by one of the most important international waterways, the Persian Gulf and the Sea of Oman [10] that need marine medicine education, and on the other hand, the importance of maritime medicine as a growing and less known field, as well as the lack of consensus in the world on the content of the maritime medicine content, the present study was conducted with the purpose of development the of marine medicine syllabus for medical sciences student.

## Methods

### Study design and ethical approval

**Fig. 1:**
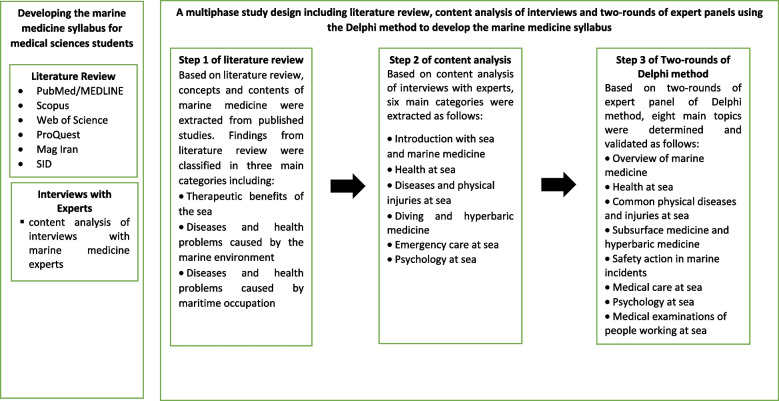
Multiphase study design for developing the marine medicine syllabus for medical sciences student

This multiphase design study was conducted to develop the marine medicine syllabus for medical sciences students, which includes three phases. The first phase was a literature review to identify concepts and contents of marine medicine in published studies. The second phase includes a content analysis of interviews with marine medicine experts. The third phase was a Delphi method that includes two consecutive rounds to obtain consensus from the marine medicine expert panel. The graph of the study is shown in Fig. 1.

This research has been approved by the Ethics Committee of Baqiyatullah University of Medical Sciences (IR.BMSU.REC.1399.006) and conducted in Tehran, Iran from 2019 to 2022.

### First Phase: Literature review

#### Design

At this phase, a literature review was used to investigate the main subjects of marine medicine, its areas and important issue in this field in published studies.

#### Search strategy

The searches were conducted in the Web of Knowledge, Pubmed and Scopus and the Iranian databases SID and Magiran databases. The search terms include “marine”, “maritime”, “sea”, “seafarer”, “navy”, “naval”, “sailor”, “diving” and “medicine”, “medical”, “health”, “treatment”, “care”, “disease”, “sick”, “ill”, “disorder”, “poison” and “toxin”. The reference lists of articles were also reviewed using forward and backward citation tracking to identify other eligible documents. The search was limited to human studies between 1990 to 2021.

#### Inclusion and exclusion criteria

All studies that contained dimensions or concepts related to marine medicine were included in the study, regardless of the study design. Exclusion criteria included not being related to marine medicine, non-English language, and lack of access to the full text.

#### Study selection

**Fig. 2:**
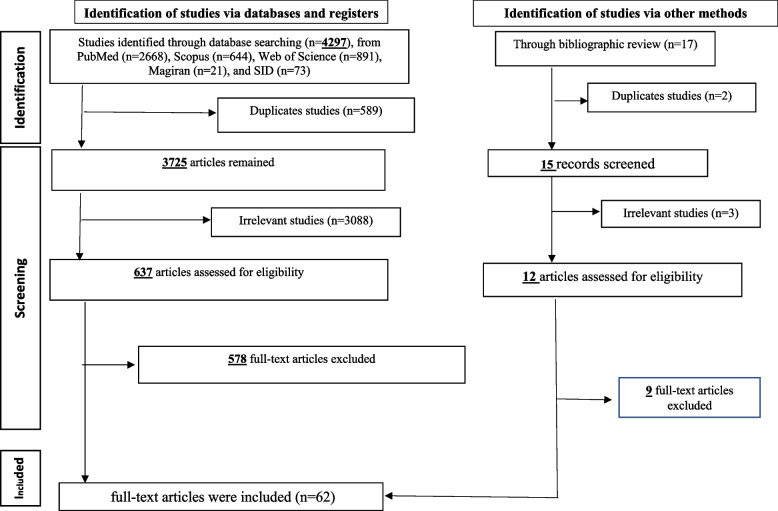
Flowchart of reviews process which include searches of databases and bibliography review according to PRISMA 2020

In the initial search by two researchers, 4314 possible articles related to marine medicine were found. A total of 589 studies were excluded due to being duplicates. Duplicate studies mean studies with the same title, names of authors and published journals. The title and abstract of 3725 studies were assessed and 3088 studies whose title and abstract were not related were excluded from the study. The full text of 637 studies was reviewed, of which 575 articles were excluded due to irrelevance or duplicate information. The remaining 62 studies were used to extract topics and concepts related to marine medicine. The flowchart of reviews process is shown in Fig. 2.

#### Data extraction

Two researchers (FH and AVA) independently examined the full text of the remaining selected papers for extracting data. All the data related to marine medicine that could be used in the main and sub topics of the marine medicine course were extracted. Any ambiguities or disagreements between the evaluators were resolved through discussion and consensus by a third person.

### Second Phase: Content analysis

#### Design

At this stage, a qualitative content analysis research method was conducted. Qualitative content analysis is the analysis of the content of narrative data, and it is a flexible method to identify prominent subthemes and patterns among themes. In the conditions of lack of enough knowledge about a phenomenon (such as marine medicine) or knowledge fragmentation, the inductive approach is recommended [11] to explain and interpret the data and elaborate the dominant and major themes of participants’ experiences [12].

#### Participants

The study participants consisted of experts and those who have experience and knowledge about marine medicine. The participants were recruited in the study using a purposeful sampling method. Also, maximum variation in sampling was considered in terms of age, working experience, degree and working field. Inclusion criteria were having experience in marine medicine, having a master's degree or doctorate in the field of medical sciences, the ability to express experiences and desire to participate in the study. The exclusion criterion was the unwillingness to continue the study. Sampling continued until data saturation was reached.

#### Data collection

Semi-structured interviews were used to collect the data. The research setting was the workplace of the participants, which the participants chose based on their preference and the researcher tried to help the participants express their experiences of marine medicine easily and freely by visiting at the chosen time of the participants and creating a safe environment and gaining their trust. The researcher obtained written informed consent from the patients before the interviews. The research questions were extracted by the discussion of the research team. The questions were open ended, neutral, clear, and relevant to the objective of the study. Four members of research team were working in the field of marine medicine which ensured the relevance of the interview questions to the research question and six members of the research team had the experience of conducting previous research interviews, which ensured the accuracy of the questions.. The Interview started with an grand tour question: what is marine medicine? Based on the answers, the interviews continued with probing and follow-up questions. All nonverbal behaviors, facial expressions and eye contact were paid attention during the interview. The duration of the interviews was between 43–72 min and an average of 56 min based on the physical and psychological conditions of the participants and the quality of their responses to the questions. All interviews were audiotaped and transcribed verbatim.

#### Data analysis

The information obtained from the interviews was analyzed by conventional content analysis with Geranheim's method. This method includes the following steps: 1-Writing down the interviews 2-Choosing the unit of analysis 3- Getting a general view of the entire interview by reading the units of analysis 4- Reading word by word and line by line and coding each meaningful unit (obtaining primary codes) of related concepts 5- Placing similar codes in a subcategory by constantly comparing codes. 6- Collecting similar subcategories in a category and naming them [13].

#### Trustworthiness

In this study, Guba and Lincoln's criteria were used to increase trustworthiness, which are known as the gold standard. For this purpose, long-term involvement with the data and spending enough time to collect and analyze the data were used. Also, all the interviews were conducted under the supervision of the supervisor, and during the data analysis, the codes and categories that emerged were presented to the supervisors and advisors to review the cases and present and apply their revisions. To increase reliability, all documents and evidence related to the data were securely stored to help readers and external observers to trust the data. Also, a number of coded interviews along with the extracted categories were given to other researchers familiar with qualitative research methods to check the accuracy of coding and classification. In order to increase transferability, the researcher tried to provide in-depth and rich descriptions of the research context and word-for-word quotes of the participants in order to increase the fit of the theory with the context under study.

The found topics in the literature review and content analysis of interviews were combined and formed the initial draft of the marine medicine syllabus, which was validated with the Delphi method in the next phase.

### Third Phase: Delphi method

#### Design

In the third phase, a Delphi method was used to obtain the consensus of experts in the syllabus of marine medicine. Delphi is a valid and scientific method to provide comprehensive and reliable information about a controversial issue or to reach a consensus on an issue, which is done through quantitative and qualitative processes [14]. The purpose of this method is to make an expert judgment about a question. This method is based on the assumption that a group of experts with different attitudes create more valid results than the judgment of one person, even if he is the best in his field [15]. One of the applications of the Delphi method in health care is in the field of education and curriculum development [16].

The Delphi method requires at least two rounds, and if the first round is open-ended, the required number of rounds is three [16]. In this research, the initial draft of the marine medicine syllabus was compiled in the previous phases, and there was no need to do the first round, therefore, this study was conducted in two rounds.

#### Participants

The Delphi panel consisted of 18 experts from health system policy makers, managers, university educators, health care providers and researchers who were well informed and experienced in the field of the marine medicine. Inclusion criteria were having at least five years work experience in marine medicine field, having a master's degree or doctorate in the field of medical sciences and desire to participate in the study. The exclusion criterion was not completing the questionnaire and unwillingness to continue the study.

#### Consensus

The main goal of the Delphi method is to reach a consensus [17]. Consensus should be determined before starting Delphi [18]. The most common method of reporting consensus is the percentage of agreement, which is defined as a proportion of participants who agree with a certain rating range [19]. The consensus is not fixed [20] and has been determined from 51 to 100% in different studies [21]. Like many studies [22–28], we also considered the cut-off point of 80% agreement as reaching consensus, and the main and sub topics that after each round of Delphi, by less than 80% of experts obtain very agree or agree, were removed from the syllabus.

#### Instrument

At first, the researchers contacted the experts and obtained their consent to participate in the study. Then the Delphi questionnaire was sent to them. The Delphi questionnaire was structured from concepts found from previous phases for a rating on a 5-point Likert scale (1-strongly disagree, 2-disagree, 3-neutral, 4-agree, 5-strongly agree). The face validity of the questionnaire was confirmed by faculty members. Interrater reliability between panelists in first round was estimated based on the Intraclass Correlation Coefficient and 0.944 Cronbach’s alpha indicated an excellent interrater agreement. Intraclass correlation coefficient (ICC) is a standard tool to determine the interrater reliability of more than two raters based on interval scaled data [29]. The five-point rating scale used in this study is interval-scaled. Interrater reliability between panelists in the second round was estimated based on the Intraclass Correlation Coefficient and 0.810 Cronbach’s alpha indicated a good interrater agreement.

#### Data analysis

After the completion of the first round of Delphi, items that had less than 80% consensus between the panelists were removed from the syllabus and topics suggested by the experts were added to the list for judging in the second round. In the second round of Delphi, topics that achieved 80% or above consensus and suggested topics consisted of the second-round questionnaire and was given to the expert panel. After the completion of the second round, the items that had less than 80% consensus among the participants were removed and the remaining topics formed the final syllabus of the marine medicine.

## Results

### Finding of the literature review

Findings from the literature review were categorized into three main categories including the therapeutic benefits of the sea category consisted of thalassotherapy and sea therapeutic products subcategories, diseases and health problems caused by the marine environment category consisted of diseases and health problems caused by marine creatures, diseases and health problems caused by diving and disease and health problems caused by marine toxins and pollution subcategories, and diseases and health problems caused by maritime occupation category consisted of diseases and health problems related to safety, diseases and health problems related to physical health and diseases and health problems related to mental health subcategories.

### Finding of the qualitative content analysis

In this study, 12 experts in the marine medicine field were interviewed. They had 38–67 years old, 7–46 years of work experience, master's or doctoral degrees, and were working in the cities of Tehran, Shiraz, Bandar Abbas and Bushehr of Iran. The participants were policymakers of the health system, managers, university educators, health care providers and researchers who had knowledge and experience in marine medicine fields. The Demographic information of participants in the interviews are shown in Table 1.

**Table 1:** Demographic information of participants in the interviews

Participants Numbers	Age (Year)	Work Experiences (Year)	Degree	Working Field
1	67	46	Doctoral	Policy making
2	51	31	Doctoral	Management
3	57	34	Doctoral	Policy making
4	57	30	Doctoral	Education
5	56	32	Doctoral	Management
6	41	26	Doctoral	Clinical
7	44	18	Doctoral	Clinical
8	53	22	Master	Education
9	48	27	Master	Education
10	51	25	Doctoral	Management
11	59	24	Doctoral	Research
12	38	7	Doctoral	Clinical

Findings from the qualitative content analysis were categorized into six categories include introduction to the sea and marine medicine consisted of introduction to the sea and introduction to the marine medicine subcategories, health at sea category consisted of importance of hygiene at sea, food health, sleep health, sea travel health and environmental health sub categories, diseases and physical injuries at sea category consisted of specific diseases of the sea, diseases and injuries caused by dangerous sea creatures, diseases and injuries caused by maritime occupation, diseases and injuries caused by naval warfare, and tropical diseases subcategories, diving and hyperbaric medicine category consisted of diving and hyperbaric medicine subcategories and emergency care at sea consisted of search and rescue, nursing and triage, cardiopulmonary resuscitation at sea and relief and transport during emergency incidents at sea subcategories and psychology at sea category consisted of depression, anxiety, addiction, factitious illnesses, job burnout and suicide subcategories.

### Finding of the Delphi method

Also, the Delphi panel consist of 18 experts including policymakers of the health system, managers, university educators, health care providers and researchers who had knowledge and experience in marine medicine fields. The panel experts had 38 to 60 years, 11 to 40 years of work experience, master's or doctorate degrees in Tehran, Shiraz, Bushehr and Bandar Abbas cities of Iran. Table 2 shows the demographic information of the Delphi expert panel.

**Table 2:** Demographic information of the Delphi expert panel

Participants Numbers	Age (Year)	Work Experiences (Year)	Degree	Working Field
1	38	19	Master	Clinical
2	55	36	Doctoral	Education
3	44	13	Doctoral	Education
4	50	30	Doctoral	Education
5	38	15	Master	Clinical
6	44	26	Master	Research
7	60	40	Doctoral	Policy making
8	56	34	Doctoral	Research
9	51	31	Doctoral	Management
10	57	34	Doctoral	Policy making
11	58	35	Doctoral	Research
12	41	11	Doctoral	Clinical
13	43	27	Master	Research
14	38	19	Doctoral	Management
15	45	36	Doctoral	Education
16	49	29	Master	Management
17	45	26	Master	Management
18	54	30	Doctoral	Education

### Delphi first round

The main and sub topics extracted in the literature review and content analysis were combined and formed the first round Delphi questionnaire. This topics include overview of marine medicine, therapeutic uses of the sea, health at sea, common physical diseases and injuries at sea, diving and hyperbaric, emergency at sea and psychology at sea. After the first round the therapeutic uses of the sea main topic obtains 22.3%, thalassotherapy sub topic obtains 22.3%, sea therapeutic products sub topic obtains 33.5%, importance of health at sea sub topic obtains 66.7% and importance of psychology at sea sub topic obtains 55.7% of consensus and removed from syllabus list. Also, some topics were suggested by experts at this round that added to the list of topics.

### Delphi second round

In the second round, one of the experts don’t complete the questionnaire and this round was conducted with 17 expert panels. Delphi second round questionnaire main topics were similar to the first round except the therapeutic uses of the sea main topic was removed.. After the second round, only the maritime convention subtopic been removed from the syllabus because of obtained 64.7% consensus and other main topics and subtopics obtained above 80% consensus and were confirmed. Percents of consensus of the Delphi expert panel in rounds 1 and 2 are shown in Table 3. The topics extracted in each phase are shown in Table 4.

**Table 3:** Percent of consensus of Delphi expert panel in round 1 and 2

Main-topics	Sub-topics	Consensus (Round 1)	Consensus (Round 2)
**Overview of marine medicine**		88.9%	94.1%
	Introduction to the sea	83.3%	
	Introduction to the seas and coasts of Iran		94.1%
	Introduction to the marine medicine	83.3%	94.1%
	Introduction to marine rescue organizations		82.4%
	International health rules and regulations in the marine domain		88.24%
			64.7%
**Therapeutic benefits of the sea**		22.4%	
	Thalassotherapy	22.4%	
	sea therapeutic products	27.8%	
**Health at sea**		100%	100%
	Importance of health at sea	66.7%	
	Personal health		94.1%
	Food health	100%	100%
	Sleep health	94.4%	94.1%
	Sea travel health	94.4%	94.1%
	Environmental health	100%	100%
	Occupational health		94.1%
**Physical diseases and injuries at sea**		100%	100%
	Diseases specific to the sea	100%	100%
	Diseases and injuries caused by dangerous sea creatures	94.4%	100%
	Diseases and injuries caused by marine toxins	88.9%	94.1%
	Diseases and injuries caused by marine occupation	94.4%	94.1%
	Injuries and damages caused by naval combat	100%	100%
	Infectious and tropical diseases	83.3%	88.24%
**Subsurface medicine and hyperbaric medicine**		100%	100%
	Introduction to the submarine		94.1%
	Introduction to the diving	100%	88.9%
	Common diseases and complications in diving		100%
	Diving medical assistant		82.4%
	Hyperbaric medicine	100%	100%
**Emergency care at sea**		100%	
**Safety action in marine incidents**			100%
	Seeking help at sea		88.2%
	Survival at sea		88.2%
	Search and rescue	100%	100%
	Relief and transfer	100%	100%
	Marine terrorism and pirates		82.36%
**Medical care at sea**			100%
	Familiarity with floating infirmary		100%
	Medicine and medical equipment standards in sea missions		100%
	Nursing and triage	100%	100%
	Cardiopulmonary resuscitation in water	100%	100%
**Psychology at sea**		94.4%	94.1%
	Importance of psychology at sea	55.6%	
	Depression	88.9%	94.1%
	Anxiety	94.4%	94.1%
	Addiction	83.3%	88.2%
	Factitious disorder	83.3%	82.4%
	Burnout	100%	100%
	Suicide	88.9%	94.1%
	Psychological, social and cognitive problems of the families of the naval personnel		94.1%
**Medical examinations of people working at sea**			94.1%
	Pre-employment medical examinations of people for working at sea		94.1%
	Periodic medical examinations of people working at sea		94.1%
	Diving license examinations		94.1%

**Table 4:** The topics extracted in each phase

Main topics	Sub topics	Literature Review	Content analysis	Delphi
**Therapeutic benefits of the sea**		^*^		
	Thalassotherapy	^*^		
	Sea therapeutic products	^*^		
**Diseases and health problems caused by the marine environment**		^*^		^*^
	Diseases and health problems caused by marine creatures	^*^		^*^
	Diseases and health problems caused by diving	^*^		^*^
	Disease and health problems caused by marine toxins and pollution	^*^		^*^
**Diseases and health problems caused by the maritime occupation**		^*^		^*^
	Diseases and health problems related to safety	^*^		^*^
	Diseases and health problems related to physical health	^*^		^*^
	Diseases and health problems related to mental health	^*^		^*^
**Introduction to the sea and marine medicine**			^*^	^*^
	Introduction to the sea		^*^	
	Introduction with marine medicine		^*^	^*^
	Introduction to the seas and coasts of Iran			^*^
	Introduction to marine rescue organizations			^*^
	International health rules and regulations in the marine domain			^*^
**Health at sea**			^*^	^*^
	Importance of health at sea		^*^	
	Food hygiene		^*^	^*^
	Sleep hygiene		^*^	^*^
	Sea travel health		^*^	^*^
	Environmental hygiene		^*^	^*^
	Personal health			^*^
	Occupational health			^*^
**Diseases and physical injuries at sea**			^*^	^*^
	Specific diseases of the sea		^*^	^*^
	Diseases and injuries caused by dangerous sea creatures		^*^	^*^
	Diseases and injuries caused by maritime occupation		^*^	^*^
	Diseases and injuries caused by naval warfare		^*^	^*^
	Tropical disease		^*^	^*^
**Diving and hyperbaric medicine**			^*^	^*^
	Diving		^*^	^*^
	Hyperbaric medicine		^*^	^*^
	Introduction to the submarine			^*^
	Common diseases and complications in diving			^*^
	Diving medical assistant			^*^
**Emergency care at sea**			^*^	
	Search and rescue		^*^	^*^
	Nursing and triage		^*^	^*^
	Cardiopulmonary resuscitation at sea		^*^	^*^
	Relief and transfer		^*^	^*^
**Safety action in marine incidents**				^*^
	Seeking help at sea			^*^
	Survival at sea			^*^
	Marine terrorism and pirates			^*^
**Medical care at sea**				^*^
	Familiarity with floating infirmary			^*^
	Medicine and medical equipment standards in sea missions			
**Psychology at sea**			^*^	^*^
	Importance of psychology at sea		*	
	Depression		^*^	^*^
	Anxiety		^*^	^*^
	Addiction		^*^	^*^
	Factitious diseases		^*^	^*^
	Job burnout		^*^	^*^
	Suicide			^*^
	Psychological, social and cognitive problems of the families of the naval personnel		^*^	^*^
**Medical examinations of people working at sea**				^*^
	Pre-employment medical examinations of people for working at sea			^*^
	Periodic medical examinations of people working at sea			^*^
	Diving license examinations			^*^

^*^This main-topic or sub-topic was extracted in this phase

### Final main topics and sub topics

In this study, eight main topics and forty sub topics of the marine medicine syllabus were found, which are summarized in Table 5.

**Table 5:** Final main topics and sub topics of marine medicine syllabus

Course	Main topics	Sub topics
**Marine Medicine**	**Overview of marine medicine**	Introduction to the seas and coasts of Iran
		Introduction to the marine medicine
		Introduction to marine rescue organizations
		International health rules and regulations in the marine domain
	**Health at sea**	Personal health
		Food health
		Sleep health
		Sea travel health
		Environmental health
		Occupational health
	**Common physical diseases and injuries at sea**	Diseases specific to the sea
		Diseases and injuries caused by dangerous sea creatures
		Diseases and injuries caused by marine toxins
		Diseases and injuries caused by marine occupation
		Injuries and damages caused by naval combat
		Infectious and tropical diseases
	**Subsurface medicine and hyperbaric medicine**	Introduction to the submarine
		Introduction to the diving
		Common diseases and complications in diving
		Diving medical assistant
		Hyperbaric medicine
	**Safety action in marine incidents**	Seeking help at sea
		Survival at sea
		Search and rescue
		Relief and transfer
		Marine terrorism and pirates
	**Medical care at sea**	Familiarity with floating infirmary
		Medicine and medical equipment standards in sea missions
		Nursing and triage
		Cardiopulmonary resuscitation in water
	**Psychology at sea**	Depression
		Anxiety
		Addiction
		Factitious disorder
		Burnout
		Suicide
		Psychological, social and cognitive problems of the families of the naval personnel
	**Medical examinations of people working at sea**	Pre-employment medical examinations of people for working at sea
		Periodic medical examinations of people working at sea
		Diving license examinations

### Overview of marine medicine

At first, it is necessary for all students to get a general picture of marine medicine and the differences and similarities of this field with general medicine. This main topic includes introduction to the seas and coasts of Iran, introduction to the marine medicine, introduction to marine rescue organizations and international health rules and regulations in the marine domain.

### Health at sea

Treatment of diseases at sea is very challenging. Therefore, we must use all necessary measures and facilities to prevent diseases and injuries in the sea. Therefore, health at sea is very important and should be given a lot of attention. The aim of health at sea is the improving and providing the highest possible level of physical, mental and social health for persons at sea. This main topic includes personal health, food health, sleep health, sea travel health, environmental health and occupational health at sea.

### Common physical diseases and injuries at sea

The most important main topic in the marine medicine course is to acquaint the students with common diseases and physical injuries at sea. Some of these diseases are not seen on land and others are common between land and sea, but treatment in sea is challenging due to the special limitations of the sea. Students should be well aware of how to manage and treat these diseases. This main topic includes diseases specific to the sea, diseases and injuries caused by dangerous sea creatures, diseases and injuries caused by marine toxins, diseases and injuries caused by marine occupation, injuries and damages caused by naval combat, infectious and tropical diseases.

### Subsurface medicine and hyperbaric medicine

Working at sea includes not only the surface but also the subsurface such as diving and submarine. The subsurface is a very specialized area that can cause special and important problems for people, which can lead to unfortunate consequences if not proper and timely be treated. This main topic includes introduction to the submarine, introduction to the diving, common diseases and complications in diving, diving medical assistant and hyperbaric medicine sub topic.

### Safety action in marine incidents

Management of emergency situations at sea demands the seafarer to know and understand a set of rules and procedures and comply them. The aim of teaching safety action in marine incidents is to provide the student with the skills and knowledge required to react appropriately in an emergency situation. This main topic includes seeking help at sea, survival at sea, search and rescue, relief and transfer, marine terrorism and pirates.

### Medical care at sea

Some aspects of medical care at sea differ from land. This difference is due to both the unique problems that may arise at sea and the limited medical facilities and equipment that exist at sea. Therefore, students should be well acquainted with the medical equipment available at sea and some medical procedures different from land, such as how to do CPR in water. This main topic includes familiarity with the floating infirmary, medicine and medical equipment standards in sea missions, nursing and triage and cardiopulmonary resuscitation in water.

### Psychology at sea

Special conditions of the sea provide a basis for the occurrence of mental disorders in people. The people in the sea are isolated and everything they see is water and there is no land around them, and this causes anxiety and depression. The root of anxiety is the lack of control over the situation and floating vehicles are moving very fast and so the feeling of mastery at sea is very low and the stress hormones level in the sea is much higher than normal that cause several psychological problems. This main topic includes depression, anxiety, addiction, factitious disorder, burnout, suicide and psychological, social and cognitive problems of the families of the naval personnel.

### Medical examinations of people working at sea

Disease and injuries at sea can be very troublesome, and one of the ways to prevent them is to select the proper persons to work at sea, which is done through medical examinations. The aim of the medical examination is to ensure that the seafarer being examined is medically fit to perform his or her routine and emergency duties at sea and is not suffering from any medical condition likely to be aggravated by service at sea, to render him or her unfit for service or to endanger the health of other persons on board. This main topic includes pre-employment medical examinations of people for working at sea, periodic medical examinations of people working at sea and diving license examinations.

## Discussion

This study investigated the marine medicine syllabus for medical sciences student education. The findings showed that the marine medicine syllabus should include an overview of marine medicine, health at sea, common physical diseases and injuries at sea, subsurface medicine and hyperbaric, safety action in marine incidents, medical care at sea, psychology at sea and medical examinations of people working at sea main topics and their sub topics.

The information obtained about the topics of marine medicine in the world is limited and, in some countries, only the full course of marine medicine is taught. The Verna Medical University of Bulgaria organizes an elective course in marine medicine. The first marine medicine training course was held at this university in 2008–2009. The main theoretical topics of this course included marine physiology, diving medicine, marine toxicology, emergency aid at sea, rescue activity at sea, marine psychology, hyperbaric oxygen therapy, marine expertise and telemedicine. The practical classes of this course also included the organization of marine services, medical care of divers, diving and hyperbaric medicine, and marine toxicology [30]. The topics reported in this full course have a lot of overlap with the topics of the present study and diving medicine, marine toxicology, emergency aid at sea, rescue at sea, marine psychology, hyperbaric oxygen therapy and medical care of divers topics are also present in the present study.

In Singapore, the course of medical care on ships is organized by the Singapore maritime academy. This course is popular both within Singapore and in the region including Malaysia, Philippines, Thailand, Sri Lanka, India and Pakistan. This course includes the topics of medical emergency management, telemedicine recommendations, tropical and infectious diseases, general principles and rules in keeping records, medical care for rescued people, treatment of poisoned patients and risks from toxicological incidents, death symptoms and medical research, alcoholism and substance abuse, wounds, wound healing and infections, wound and burn dressings and area debridement and suturing procedures, management of surgical emergencies, nursing techniques, gynecology, pregnancy and childbirth, dental care, sexually transmitted diseases, environmental control on the ship deck, transportation of sick and injured people and basic cardiopulmonary resuscitation [4]. Medical emergency management, tropical and infectious disease, medical care for rescued people, treatment of poisoned patients and risks from toxicological incidents, alcoholism and substance abuse, wounds, wound healing and infections, wound and burn dressings and area debridement and suturing procedures, nursing techniques, environmental control on the ship deck, transportation of sick and injured people and basic cardiopulmonary resuscitation topics reported in this study have overlap in some way with the topics found in our study. Although some topics are different in both studies which are due to the difference in the type of marine medicine education, and we have considered marine medicine as a lesson and have included the general topics related to it in the syllabus, but in the mentioned study, marine medicine has been considered as a full course and more specific topics are included. Also, different experiences and educational needs in different countries are also one of the reasons that made the educational topics of different countries are not completely the same.

The University of Bergen in Norway offers a master’s degree in maritime medicine. In this course, maritime medicine is defined as a field of medicine including all aspects of work at sea, such as public health, occupational medicine, general medicine, emergency medicine, remote medicine, etc. The main topics of this course include a general overview of maritime medicine, an overview of specific occupational health related challenges in the most important export industries in Norway, a basic introduction to principles of selection medicine, an overview of Norwegian maritime rescue services, an introduction to various tools for telemedicine treatment, their limitations, advantages and disadvantages [31]. The general overview of maritime medicine, an overview of specific occupational health and maritime rescue services main topics are overlap with main topics found in the present study, although in this course, most topics are taught specifically for Norway. Also, remote medicine has an important role in this marine medicine course, which is not present in the topics found in the present study due to different educational needs.

The University of Cádiz, Spain, established a master’s degree in marine health in 2015–2016 by the faculty of medicine in collaboration with the Spanish society of marine medicine and the international society of marine health. The content of this program in the first semester were history, laws and regulations, statistics and epidemiology, evidence-based marine medicine. In the second semester, the content were national and international organizations, medical care and preventive maritime medicine (including telemedicine, on-board medicine, physical fitness medical tests), working conditions and risk prevention, accident medicine, tropical medicine, maritime and port medicine, poison science, health, survival and rescue in the water. The 3^rd^ and 4^th^ semesters, the content of programs included underwater and hyperbaric medicine, water sports medicine, physical fitness tests for professional divers and water athletes [32]. The laws and regulations, national and international organizations, medical care and preventive maritime medicine, working conditions and risk prevention, accident medicine, tropical medicine, poison science, health, survival and rescue in the water, underwater and hyperbaric medicine, physical fitness tests for professional divers topics reported in this study have overlap with the topics found in the present study. Although the difference in the type of marine medicine education and the difference in educational opportunities as well as the different experiences and educational needs of Iran and Spain lead to some of the educational topics of the present study are different from the mentioned study.

Also, many of the necessary knowledge and skill for ship doctors reported in McCarthy et al. [6], Bygvraa et al. [33], Seidenstuecker and Neidhardt [34], and Bobdey et al. [35] studies overlap with the topics found in the present study.

## Conclusion

Marine medicine is an extent and specialized medical field which has been neglected and it is necessary to teach this lesson with the syllabus found in the present study to medical sciences students in the general course. Marine medicine syllabus should include overview of marine medicine main topic consist of introduction to the seas and coasts of Iran, introduction to the marine medicine, introduction to marine rescue organizations, international health rules and regulations in the marine domain sub topics and health at sea main topic consist of personal health, food health, sleep health, sea travel health, environmental health, occupational health sub topics and common physical diseases and injuries at sea main topic consist of diseases specific to the sea, diseases and injuries caused by dangerous sea creatures, diseases and injuries caused by marine toxins, diseases and injuries caused by marine occupation, injuries and damages caused by naval combat, infectious and tropical diseases sub topics and subsurface medicine and hyperbaric medicine main topic consist of introduction to the submarine, introduction to the diving, common diseases and complications in diving, diving medical assistant, hyperbaric medicine sub topics and safety action in marine incidents main topic consist of seeking help at sea, survival at sea, search and rescue, relief and transfer, marine terrorism and pirates sub topics and medical care at sea main topic consist of familiarity with floating infirmary, medicine and medical equipment standards in sea missions, nursing and triage, cardiopulmonary resuscitation in water sub topics and psychology at sea main topic consist of depression, anxiety, addiction, factitious disorder, burnout, suicide, psychological, social and cognitive problems of the families of the naval personnel sub topics and medical examinations of people working at sea main topic consist of pre-employment medical examinations of people for working at sea, periodic medical examinations of people working at sea, diving license examinations.

## Data Availability

The data that support the findings of this study are not publicly available due to their containing information that could compromise the privacy of research participants but are available from the corresponding author.
